# Intestinal STING‐(Over)activation—A Contributing Factor to Food Allergies?

**DOI:** 10.1111/all.70345

**Published:** 2026-04-18

**Authors:** Sophie Goebel, Hannah Ruth Schiller

**Affiliations:** ^1^ Department of Paediatrics, Division of Pneumology, Allergology, Infectious Diseases and Gastroenterology Goethe University Frankfurt Frankfurt am Main Germany; ^2^ Allergology Research Section, Allergology Division Paul‐Ehrlich‐Institut Langen Germany

**Abbreviations**
APCs: antigen presenting cells
CCR7: C‐C chemokine receptor type 7
CDNs: cyclic dinucleotides
cGAS: cyclic GMP‐AMP synthase
DC: dendritic cell
ILC3: innate lymphoid cell type 3
pTregs: peripherally induced antigen‐specific regulatory T cells
RORγt: retinoid‐related orphan receptor gamma t
sIgE: specific immunoglobulin E
STING: stimulator of interferon genes
Treg: regulatory T cell

Throughout life, the gastrointestinal immune system is confronted with a wide variety of antigens. To maintain tolerance, intestinal antigen‐presenting cells (APCs) induce peripheral antigen‐specific regulatory T cells (pTregs) to commensal microorganisms and dietary antigens [1]. A dysregulated gut tolerance is associated with immune‐related disorders, including allergies [1, 2, 3].

Antigen‐presenting innate lymphoid cells type 3 (ILC3s) expressing the transcription factor retinoid‐related orphan receptor gamma t (RORγt) have been identified as essential for inducing microbiota‐specific tolerance in the gut [1]. Zhou et al. have recently discovered that in CCD6^+^ ILC3s, the cytosolic DNA sensor stimulator of interferon genes (STING) directly sensed bacterial‐derived cyclic dinucleotides and initiated the induction of microbiota‐specific Tregs [1]. The cyclic GMP‐AMP synthase (cGAS)‐independent STING activation increased the expression of C‐C chemokine receptor type 7 (CCR7), facilitating subsequent ILC3 migration to draining lymph nodes, where they promoted microbiota‐specific Tregs [1] (Figure 1). Mechanistically, STING‐deletion increased intestinal inflammation, reduced frequencies of RORγt^+^ Tregs and altered microbiota in mice, whereas STING‐overactivation led to ILC3 death, resulting in lower Treg frequencies [1]. Consistently, increased STING activation in ILC3s and lower frequencies of ILC3s and Tregs were found in intestinal biopsies from Crohn's disease patients [1]. Therefore, STING activation in antigen‐presenting ILC3s seems crucial for intestinal immune tolerance and gut homeostasis, whilst STING‐overactivation in inflamed tissue contributes to the depletion of tissue‐protective ILC3s and thereby to disease pathogenesis.

**FIGURE 1:**
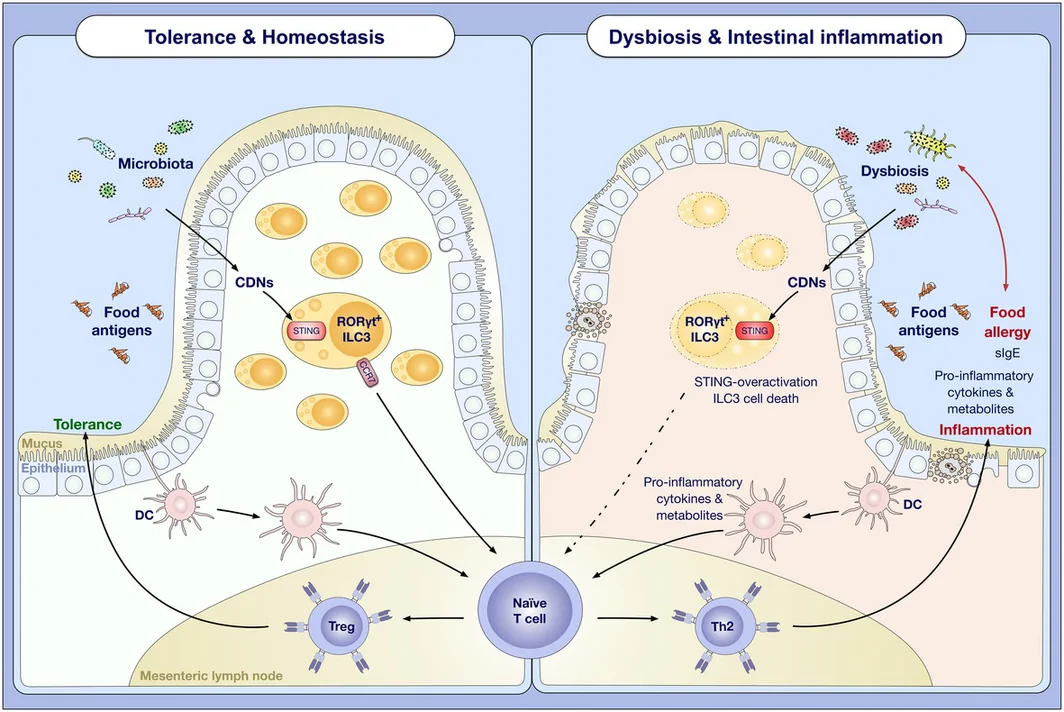
Possible contribution of STING‐(over)activation in ILC3s to food allergy. Antigen‐presenting cells (APCs), such as RORγt^+^ ILC3s and DCs, play a key role in mediating immune tolerance to intestinal microbiota and dietary antigens. Zhou et al. [1] discovered that CCD6^+^ ILC3s sense bacterial‐derived CDNs directly via the STING pathway, followed by the expression of CCR7, which facilitates subsequent ILC3 migration to draining mesenteric lymph nodes and the induction of microbiota‐specific Tregs. Conversely, STING overactivation, such as that caused by intestinal inflammation or dysbiosis, resulted in ILC3 death and reduced Treg frequencies. These findings could contribute to the development of food allergies, as gut dysbiosis, STING‐overactivation and the resulting death of ILC3s could reinforce a pro‐inflammatory environment. This could alter how RORγt^+^ APCs prime naïve T cells against dietary antigens, shifting the immune response from Treg‐mediated tolerance to Th2 responses. This could result in exacerbated intestinal inflammation and the development of food allergies, particularly during solid food introduction in infancy. CCR7, C‐C chemokine receptor type 7; CDNs, cyclic dinucleotides; DC, dendritic cell; ILC3, innate lymphoid cells type 3; RORγt, retinoid‐related orphan receptor gamma t; sIgE, specific immunoglobulin E; STING, stimulator of interferon genes; Treg, regulatory T cell.

Zhou et al.'s findings could be relevant for the development of allergies, either directly or indirectly. Recent evidence shows that in the gut, dietary antigen‐specific Tregs are primarily induced by RORγt^+^ dendritic cells, rather than ILC3s [2, 3]. However, ILC3s in the lymph nodes of the lung were described to control type 2 allergic airway inflammation [4]. In contrast, no differences were observed in circulating ILC3 levels between healthy controls and allergic patients, but an altered heterogeneity of ILC3s during allergen‐specific immunotherapy [5]. Additionally, STING‐ or cGAS‐deficient mice or mice treated with a cGAS‐inhibitor were protected against allergic type 2 airway inflammation [6]. Furthermore, STING may also affect the migration of other APCs in lymphatic tissues, where they instruct the differentiation of antigen‐specific T‐ and B‐cells [1, 5]. Therefore, the STING‐activated ILC3 axis might be implicated in allergic lung diseases, but so far, its direct involvement in food allergy remains speculative.

However, Zhou et al.'s findings could indirectly impact food allergies. Food‐allergic children exhibit altered gut microbiota compared to healthy controls [3]. Disruptions to the gut microbiome through dietary changes or antibiotics may cause a shift from anti‐ to pro‐inflammatory metabolites and cytokines [3]. By inducing STING‐overactivation and ILC3 death [1], early‐life microbiome dysbiosis could directly reinforce the pro‐inflammatory environment and thereby increase susceptibility to allergic sensitisation. During the critical ‘window of opportunity’ in infancy, solid food introduction coincides with increased frequencies of RORγt^+^ APCs and enhanced pTreg differentiation [3]. Here, the priming of naïve T cells by RORγt^+^ APCs against dietary antigens can be modulated in an inflammatory context, shifting the immune response from pTreg‐mediated tolerance to Th2 responses [2]. Thus, achieving balanced host‐microbiome interactions and counteracting a pro‐inflammatory environment during early gut maturation appears to be decisive for attaining persistent immunity [2, 3, 5]. Otherwise, STING‐mediated ILC3 death could indirectly lead to higher susceptibility to allergies by further potentiating an inflammatory environment in the gut.

To summarise, Zhou et al. have demonstrated that ILC3s sense gut microbiota through STING to induce immune tolerance [1], thereby improving our understanding of the mechanisms behind tolerance induction in the gut. STING‐overactivation could indirectly influence oral tolerance to food antigens and thus contribute to the development and maintenance of food allergies. However, further research is needed to evaluate if targeting STING could pose an approach for preventive or therapeutic strategies in food allergies.

## Funding

The authors have nothing to report.

## Disclosure

Hannah Ruth Schiller: The views expressed in this work are the personal views of the author and may not be understood or quoted as being made on behalf of or reflecting the position of the respective national competent authority, the European Medicines Agency or one of its committees or working parties.

## Conflicts of Interest

The authors declare no conflicts of interest.

## Data Availability

Data sharing not applicable to this article as no datasets were generated or analyzed during the current study.
